# The Therapeutic Effects of Bioactive Compounds on Colorectal Cancer via PI3K/Akt/mTOR Signaling Pathway: A Critical Review

**DOI:** 10.1002/fsn3.4534

**Published:** 2024-11-07

**Authors:** Kübra Demir, Rana Turgut, Selcen Şentürk, Handan Işıklar, Elif Günalan

**Affiliations:** ^1^ Institute of Graduate Education Istanbul Health and Technology University Istanbul Türkiye; ^2^ Faculty of Health Science, Department of Nutrition and Dietetics Sabahattin Zaim University Istanbul Türkiye; ^3^ Faculty of Medicine, Department of Internal Medicine Yalova University Yalova Türkiye; ^4^ Faculty of Health Science, Department of Nutrition and Dietetics Istanbul Health and Technology University Istanbul Türkiye

**Keywords:** alkaloids, colorectal cancer, polyphenols, terpenoids

## Abstract

Understanding the molecular signaling pathways of colorectal cancer (CRC) can be accepted as the first step in treatment strategy. Permanent mTOR signaling activation stimulates the CRC process via various biological processes. It supplies the survival of CRC stem cells, tumorigenesis, morbidity, and decreased response to drugs in CRC pathogenesis. Therefore, inhibition of the mTOR signaling by numerous bioactive components may be effective against CRC. The study aims to discuss the therapeutic capacity of various polyphenols, terpenoids, and alkaloids on CRC via the PI3K/Akt/mTOR pathway. The potential molecular effects of bioactive compounds on the mTOR pathway's upstream and downstream targets are examined. Each bioactive component causes various physiological processes, such as triggering free radical production, disruption of mitochondrial membrane potential, cell cycle arrest, inhibition of CRC stem cell migration, and suppression of glycolysis through mTOR signaling inhibition. As a result, carcinogenesis is inhibited by inducing apoptosis and autophagy. However, it should be noted that studies are primarily in vitro dose‐dependent treatment researchers. This study raises awareness about the role of phenolic compounds in treating CRC, contributing to their future use as anticancer agents. These bioactive compounds have the potential to be developed into food supplementation to prevent and treat various cancer types including CRC. This review has the potential to lead to further development of clinical studies. In the future, mTOR inhibition by applying several bioactive agents using advanced drug delivery systems may contribute to CRC treatment with 3D cell culture and in vivo clinical studies.

## Graphical abstract

This study discussed the therapeutic capacity of various polyphenols, terpenoids, and alkaloids on CRC via the PI3K/Akt/mTOR pathway. Each bioactive component causes various physiological processes, such as triggering free radical production, disruption of mitochondrial membrane potential, cell cycle arrest, inhibition of CRC stem cell migration, suppression of glycolysis, and induction of apoptosis and autophagy through mTOR signaling inhibition.

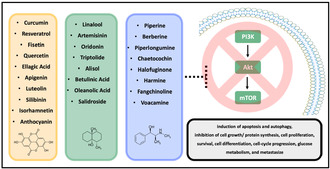

## 1 Introduction

Colorectal cancer (CRC) is one of the most common causes of cancer‐associated morbidity and mortality globally (Stefani et al. 2021; Amanpour et al. 2022; Li et al. 2014). Prostate cancer, lung cancer, and CRC account for almost one‐half (48%) of all incident cases in men. For women, breast cancer, lung cancer, and CRC account for 52% of all new diagnoses. The most significant number of deaths are from cancers of the lung, prostate, and colorectum in men and cancers of the lung, breast, and colorectum in women (Siegel et al. 2023). According to Turkish Statistical Institute data, benign and malignant tumors rank 2nd among the causes of death (15%). Among tumors, malignant tumor of the colon ranks second (7.7%) in the Türkiye (Turkish Statistical Institute 2024). Moreover, colon cancer incidence ranks 4th worldwide and 5th in terms of mortality, according to the Global Cancer Observatory data. Rectal cancer ranks 8th worldwide and 10th in terms of mortality (Ferlay et al. 2024). The burden of CRC is estimated to increase to 3.2 million new cases and 1.6 million deaths by 2040 (Morgan et al. 2023). Research on CRC prevention strategies is more abundant due to the rapid increase in middle and high‐income countries (Ramasamy et al. 2015). The significant risk factors of CRC are smoking, physical inactivity, alcohol addiction, gut dysbiosis, obesity, Type 2 diabetes, gastrointestinal diseases, aging, and poor nutrition (Haggar and Boushey 2009). Specifically, it has been shown in many studies that excessive intake of red and processed meat increases the CRC risk. Additionally, the lack of vegetables, fruits, and whole grains in the diet also triggers carcinogenesis (Edwards et al. 2010).

The survival rate in patients with advanced colon cancer is reasonably low. Despite novel treatment strategies, recovery is rare due to the recurrence of the disease. The renewal and differentiation of CRC stem cells cause novel tumor formation (Dick 2008). There are various therapeutic approaches to treating cancer at each stage of this progressive process. One of these approaches involves inhibition, through diet and pharmacological agents, of signaling pathways known to be abnormally activated in CRC. Elimination of mammalian target of rapamycin (mTOR) signaling pathway anomalies, especially seen in CRC cells, can be evaluated in this context. Thus, it is possible to effectively combat the challenges posed by the limited clinical success of existing therapeutic agents and tumor heterogeneity in various cancer types (Ersahin, Tuncbag, and Cetin‐Atalay 2015). On the other hand, different types of CRC, including sporadic (95%) and inherited (5%), are defined in the scientific literature. Sporadical CRCs appear with somatic mutation, while inherited CRCs are related to germline mutations. Different types of CRCs have different mutations in signaling pathways, including Wnt‐β‐catenin, tyrosine kinase receptors, TGFβ, Hedgehog, cell cycle checkpoints, and apoptotic pathways (Centelles 2012). Apart from that, the role of the mTOR signaling pathway has been intensely discussed in the pathogenesis of CRC in recent years (Wang and Zhang 2014).

mTOR was discovered in the early 1990s as a great protein targeted by the rapamycin agent (Sarbassov et al. 2005). Over the following decades, scientists used rapamycin to discover mTOR‐dependent processes (Guertin and Sabatini 2005). The mTOR signaling pathway is the primary regulator of cell growth and proliferation. Functional abnormalities in the mechanisms regulating this pathway are related to various non‐communicable diseases. The mTOR pathway modulates ribosome biogenesis, autophagy, and metabolism via integrating signs from nutrients, energy status, and growth factors (Sarbassov et al. 2005). Current information implies that mTOR functions through mTOR complex 1 (mTORC1) and 2 (mTORC2). mTORC1 includes mTOR, GβL (a yeast homolog of LST8), raptor, PRAS40 (proline‐rich Akt substrate 40 kDa), mLST8 (also called G‐protein β‐subunit‐like protein), and DEPTOR (containing mTOR interacting protein DEP area). mTORC2 consists of mTOR, mLST8, mSin (mammalian stress‐activated protein kinase‐interacting protein 1), DEPTOR, Rictor (rapamycin‐insensitive companion of mTOR), and protor (rictor‐observed protein, also called PRR5, proline‐rich protein 5) (Liu and Sabatini 2020; Mossmann, Park, and Hall 2018).

mTOR is a 289 kDa molecular weight serine/threonine protein kinase, central to intracellular signaling. Signal transmission in cancer cells frequently includes the overstimulation of receptor tyrosine kinases (RTKs). This process is activated in the effectiveness of three main signaling pathways. These pathways are phosphatidylinositol 3‐kinase (PI3K), protein kinase B (Akt), and mitogen‐activated protein kinase (MAPK)/Ras (Stefani et al. 2021). Moreover, mTOR is related to PI3K and Akt as the main component of cellular metabolism. The PI3K/Akt/mTOR signaling pathway is a central cell growth and proliferation regulator. Its aberrant activation has been associated with various cancers including breast, lung, gastric, renal, prostate and colorectal, and the inhibition of this pathway has been demonstrated to induce tumor regression. Moreover, dysregulation of components within this pathway is often implicated in resistance to anticancer therapies. Promising findings suggest that this pathway exerts antineoplastic effects by modulating immune system‐mediated functions, tumor suppressive signaling, oncogenic signaling regulation, angiogenesis, inflammation, and the mitigation of drug resistance (Alzahrani 2019; Tian, Li, and Zhang 2019; Glaviano et al. 2023; Ganesan et al. 2024).

**FIGURE 1:**
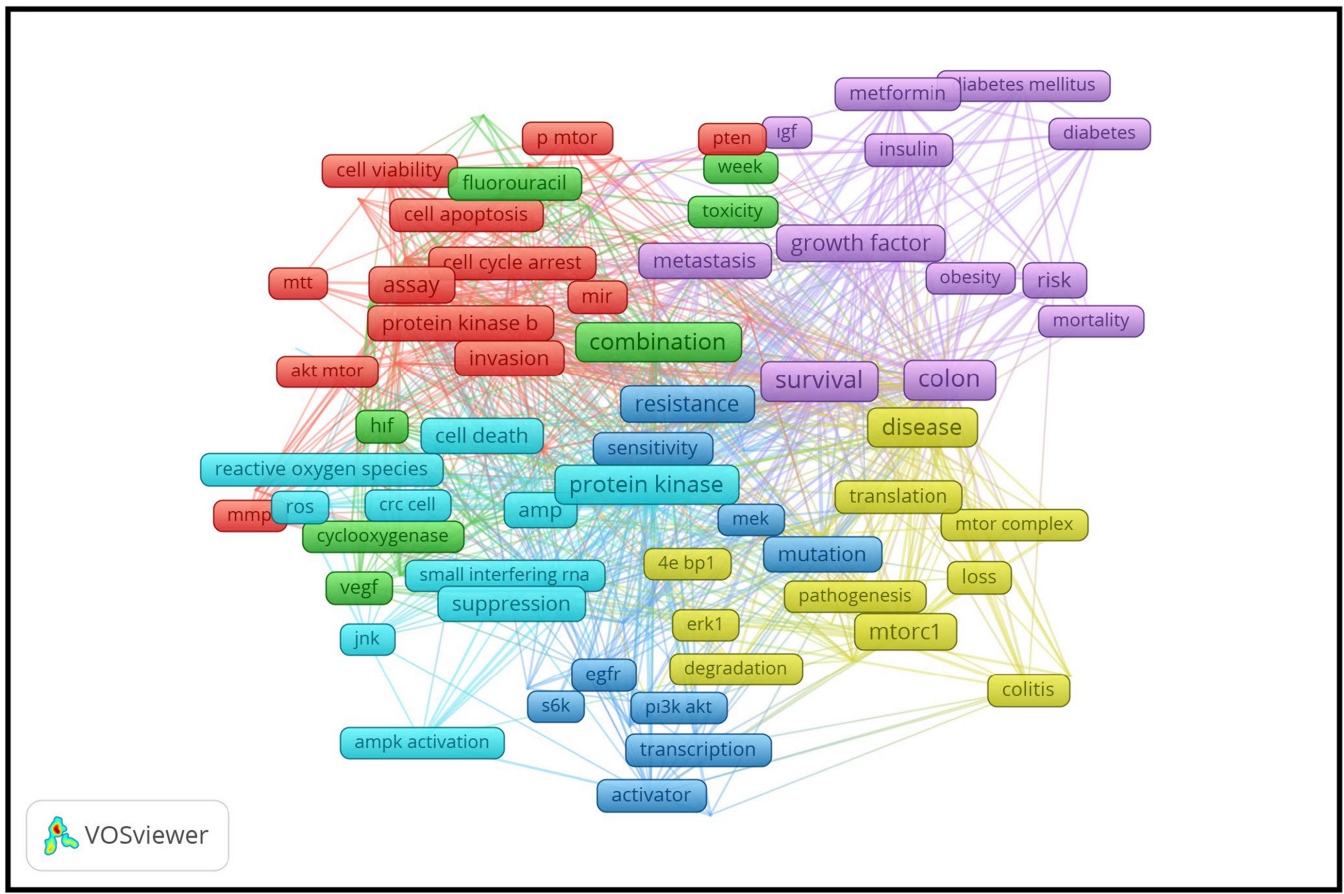
The most frequently used keywords in the articles (*n* = 395) contain the effect of the mTOR pathway on the CRC in the Web of Science database (VOSviewer (version 1.6.20, October 31, 2023)).

Targeting a single signaling pathway, such as PI3K, often results in toxicity‐induced adverse effects in patients. Therefore, the potential therapeutic efficacy of the PI3K/Akt/mTOR signaling pathway relies on combinatorial strategies. For instance, combining natural compounds with PI3K/Akt/mTOR inhibitors may mitigate toxicity, representing a significant approach to targeted cancer therapy (Yu, Wei, and Liu 2022). Apart from that, the PIK3CA gene is mutated in ~20% of CRCs, activating the PI3K/Akt/mTOR pathway. This abnormal activation significantly affects cell stress response, metabolism, and proliferation, making it an essential target in CRC treatment (Amanpour et al. 2022; Li et al. 2014; He et al. 2014; Kaur et al. 2018). In addition, the PI3K/Akt/mTOR signaling pathway also contributes to carcinogenesis, CRC stem cell survival, increased morbidity, and decreased response to drugs in CRC pathogenesis (Ebrahimi et al. 2023). In the Web of Science database, hundreds of articles have focused on the effect of mTOR signaling on the progression of CRC through cellular physiology, metabolic disorders, and mTOR components (Figure 1).

Accordingly, targeting the abnormal mTOR activation may be remarkable in the induction of the apoptosis mechanism of CRC cells (Xie, Liu, and Li 2020). In this context, various polyphenols, terpenoids, and alkaloids affect CRC progression by inhibiting the mTOR pathway. However, some bioactive compounds can accelerate cancer by activating mTOR signaling. The investigations of bioactive compounds on the mTOR signaling inhibition are necessary to evaluate as a drug target in CRC pathogenesis. Therefore, the study aims to discuss the therapeutic capacity of various polyphenols, terpenoids, and alkaloids on CRC via the PI3K/Akt/mTOR pathway in light of recent studies. Moreover, it summarizes the associations between the mTOR signaling inhibition pathway and cellular physiological outcomes.

## 2 The Potential Role of Polyphenols

**FIGURE 2:**
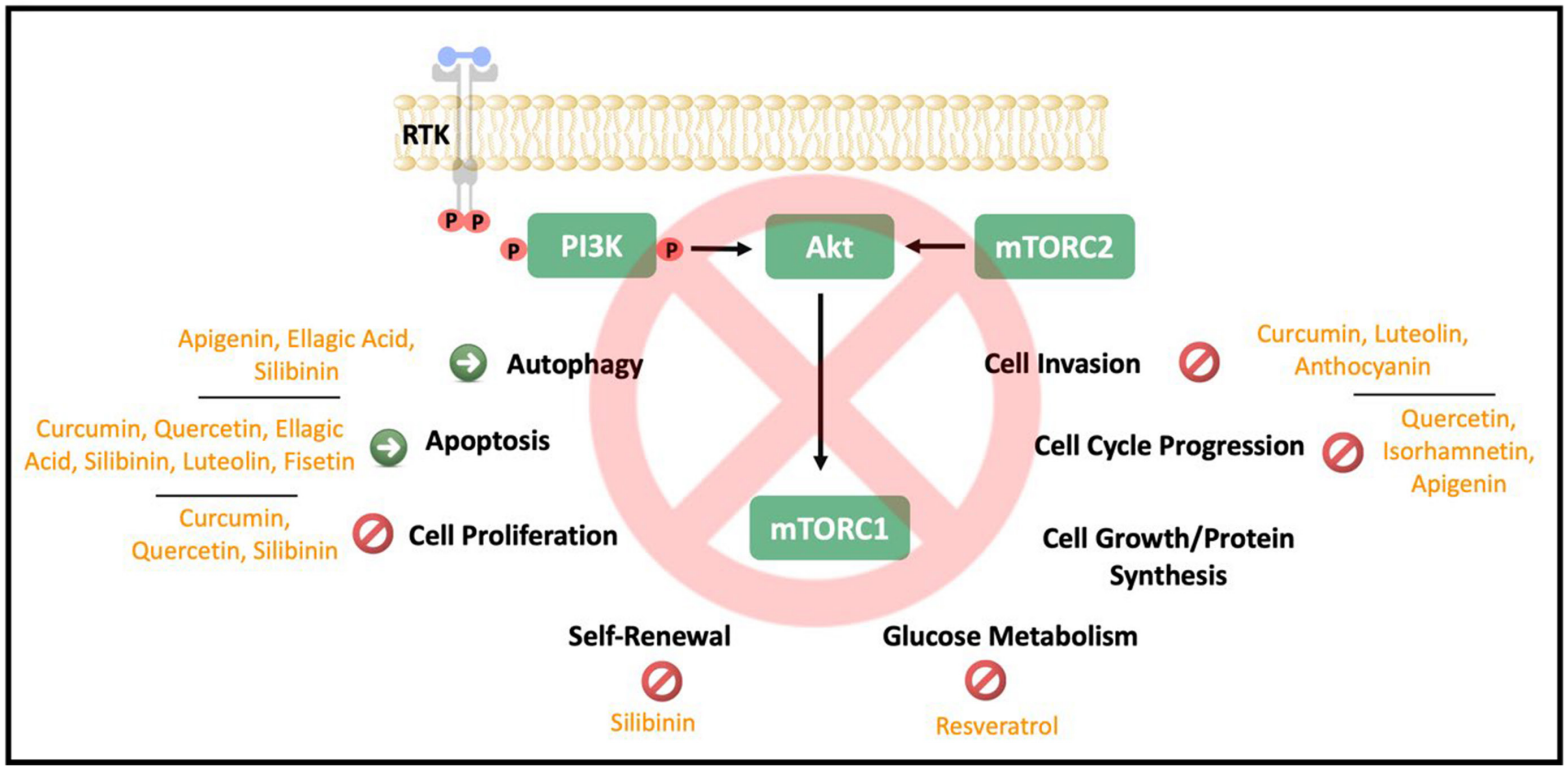
The potential cellular effects of polyphenols on CRC progression. P, Phosphate; RTK, Receptor tyrosine kinase.

The C6–C3–C6 structure polyphenols are an essential class of complex and common plant metabolites (Bravo 1998). Its components include flavonoids, flavonols, isoflavones, flavanones, anthocyanins, flavan‐3‐ols, flavones, and stilbenes. They are natural by‐products or secondary metabolites of plants. Their properties are anti‐inflammatory, antioxidant, antiallergic, antihypertensive, anticancer, and antiviral (Di Lorenzo et al. 2021). Table 1 shows polyphenols' potential therapeutic role in CRC by inhibiting the mTOR signaling. In addition, the cellular effects of polyphenols via PI3K/Akt/mTOR signaling in CRC are given in Figure 2.

**TABLE 1:** According to results of in vitro studies, the cellular effects of various polyphenols on the CRC progression via mTOR inhibition.

Bioactive compound	Study design	Effects via mTOR inhibition	References
Curcumin	Curcumin treatment on the HCT‐116 cells	Antiproliferative effects in the HCT‐116 cells	Johnson et al. (2009)
	Curcumin and curcumin analog (DMC‐BH) treatment on the HT‐29 and HCT116	DMC‐BH inhibited the proliferation and invasion and promoted the apoptosis of HCT116 and HT 29 cells. The Akt pathway activator SC79 reversed the proapoptotic effects of DMC‐BH on CRC cells, indicating that its effects are mediated by PI3K/AKT/mTOR signaling	Liu, Chen, and Bao (2023)
Resveratrol	Dose dependent treatment of OSU‐CG5 and resveratrol on the HCT‐116 and Caco‐2	Both OSU‐CG5 and resveratrol induced dose‐dependent energy restriction, suppressed glucose uptake, Akt phosphorylation and increased ER stress	Arafa et al. (2014)
	RSV‐Forskolin combination treatment on the DLD‐1 colon cancer cell line	Boosted cAMP levels, which in turn reduced the viability of the DLD‐1 colon cancer cell line	Kim et al. (2019)
Quercetin	Treatment of quercetin, doxorubicin (Dox) and combination to CD133(+) cancer stem cells of human colorectal HT29 cells	Quercetin, dox and their combination inhibited cell proliferation and induced apoptosis and G2/M arrest Combination therapy can be beneficial for drug resistance in CRC	Atashpour et al. (2015)
	Treatment of quercetin to HCT116 and HT‐29 cells	Induction of apoptosis by increased intracellular ROS through Sestrin 2/AMPK/mTOR pathway	Kim, Lee, and Kim (2013)
Ellagic acid	Treatment of ellagic acid to HCT116 cells	Induction of autophagy and apoptosis through AMPK/mTOR pathway	Ni et al. (2023)
Apigenin	Dose dependent treatment of apigenin alone and together with 5‐fluorouracil (5‐FU) for different incubation periods, on the p53 mutant HT29 human colon adenocarcinoma cell line	Alone apigenin induced cell cycle arrest via activation of caspase cascade and stimulation of apoptosis in HT29 cells	Turktekin et al. (2011)
	Dose dependent treatment of apigenin to Wnt‐stimulated P19 cancer stem cells and Wnt‐driven WiDr, HCT‐116, and SW480 colorectal cancer cells	Apigenin induced autophagy‐lysosomal degradation system of β‐catenin through inhibition of the AKT/mTOR pathway	Lin, Chen, et al. (2017)
	Treatment with lipid polymer hybrid nanoparticles of apigenin (LPHyNPs) on HCT116 cells	LPHyNPs have potential against chemoresistance and the anticancer properties through regulation of Bcl‐2, BAX, NF‐κB, and mTOR expression	Alfaleh et al. (2022)
Luteolin	Treatment of quercetin and luteolin combined with 5‐Fluorouracil (5‐FU) in the HT‐29 cell line	It exhibited the synergistic anticancer and anti‐angiogenic activity via modulation of the apoptotic pathways	Erdoğan, Ağca, and Aşkın (2022)
Silibinin	Treatment of silibinin to cancer stem‐like cells (CLSCs) enriched CRC spheroid culture system	It suppressed the colon CLSCs self‐renewal and sphere formation	Wang, Chang et al. (2012)
	Treatment with silibinin on SW480 cell	It resulted with activation of MAP2K1/2‐MAPK1/3 pathways and inhibition of PI3K‐Akt–mTOR signaling. Physiological responses of these process are increased oxidative stress, induced autophagic response, endoplasmic reticulum stress, and mimicked starvation‐like condition	Raina et al. (2013)
	Treatment with metformin or Metformin + Silibinin in human CRC cells (COLO 205)	Activation of apoptosis inducing factor on COLO 205 cells	Tsai et al. (2015)
	The combined treatment with regorafenib and silybin in SW48, SW48‐CR, HCT15 and SW480 cells	It induces synergistic anti‐proliferative and apoptotic effects and also increased the production of reactive oxygen species levels	Belli et al. (2017)
Isorhamnetin	Treatment of isorhamnetin on the HT‐29, HCT116 and SW480	It suppressed the proliferation of cells from all three cell lines, induced cell cycle arrest at the G2/M phase and suppressed cell proliferation by inhibiting the PI3K‐Akt–mTOR pathway	Li et al. (2014)
Anthocyanin	Treatment of delphinidin, one of antioxidative anthocyanins, and hydrogen peroxide on the HCT116	In the cells treated with different concentrations of delphinidin and hydrogen peroxide, the levels of p‐mTOR, p‐AKT and p‐PI3K were reduced by delphinidin while they were not affected by hydrogen peroxide	Quintos et al. (2010)

### 2.1 Curcumin

Curcumin, which belongs to the class of polyphenols with a typical yellow color, was extracted by Vogel and Pelletier in the Laboratory at Harvard College in 1815 (Chattopadhyay et al. 2004). Although curcumin is safe, its bioavailability is relatively low, according to the Food and Drug Administration (FDA) (Gupta, Patchva, and Aggarwal 2013; Prasad et al. 2014). It is nearly insoluble in water, rarely absorbs in the small intestine, and is susceptible to alkaline degradation (Hirose et al. 2022). The small amount ingested is rapidly reduced to dihydro‐, tetrahydro‐ or hexahydrocurcumin. Curcumin and its reduced metabolites are conjugated with glucuronic acid and sulfates and excreted in the urine (Lao et al. 2006). The uptake and distribution of the active compound to the site of action is an essential requirement for any biological activity. In order to overcome its poor bioavailability, several molecular strategies have been developed. These are micelle formulations of curcumin with food emulsifiers, curcumin‐cyclodextrin complexes with γ‐cyclodextrin, enhancing its water solubility by formulating liposomes or pseudo‐pyrosomes with phospholipids or nanoparticles with glycerin and gum ghatti. Combination with adjuvants such as natural turmeric oils or phytochemicals are other approaches used to inhibit intestinal efflux transporters, reduce phase II metabolism, and excretion of curcumin (Schiborr et al. 2014; Kanai et al. 2012; Yue et al. 2012; Gopi et al. 2017; Fança‐Berthon et al. 2021). The anti‐diabetic, anti‐inflammatory, antioxidant, and antiaging features of curcumin are often highlighted in the literature (Goel, Kunnumakkara, and Aggarwal 2008; He et al. 2011). Moreover, an essential therapeutic component, curcumin prevents cancer development by interacting with intracellular and extracellular molecules (Gupta, Patchva, and Aggarwal 2013; Sethi et al. 2012).

The scientific literature discusses to the effectiveness of curcumin and its derivatives in the healing of colon cancer (Kanwar et al. 2011; Lin et al. 2011; Yu et al. 2009). Curcumin suppresses the nuclear factor kappa beta (NF‐kB) in this context. NF‐kB modulates mechanisms involving angiogenesis, uncontrolled cell proliferation, apoptosis, metastasis, and resistance to chemotherapy (Johnson et al. 2009; Kunnumakkara, Anand, and Aggarwal 2008; Villegas et al. 2021). Consistently, combining curcumin with chemotherapy inhibits chemotherapy‐resistant CRC stem cells (Kanwar et al. 2011). At the same time, curcumin blocks cancer development by affecting various proteins and signaling pathways related to cell proliferation such as Akt, PI3K, mTOR, activator protein (AP1), c‐Jun N‐terminal kinase (JNK), Janus kinases‐ signal transducer and activator of transcription proteins (JAK–STAT), protein kinase C (PKC), cMYC, mitogen‐activated protein kinase (MAPK), extracellular receptor kinase (ERK), cyclin‐dependent kinases (CDKs), p53 and Wnt/ß catenin (Tsvetkov et al. 2005; Shelash Al‐Hawary et al. 2023). In particular, the therapeutic efficacy of curcumin in CRC is closely associated with the mTOR signaling pathway. The treatment of curcumin and its analogs reduces the expression levels of p‐mTOR, p‐Akt, Raptor, Rictor, p‐70S6K, and p‐4E‐BP1 proteins. The suppression of the PI3K/Akt/mTOR signaling pathway causes an antiproliferative effect on CRC cells and prevents tumor growth (Al‐Qasem et al. 2016; Liu, Chen, and Bao 2023; Moos et al. 2004; Sharma et al. 2001). Chen et al. have determined that curcumin stimulates ferroptosis by blocking the PI3K/Akt/mTOR pathway in HCT‐8 cells (Chen, Tan, and Li 2023). According to Liu, Chen, and Bao (2023) DMC‐BH, a curcumin analog, shows more substantial effects against CRC than curcumin via deactivating the PI3K/Akt/mTOR signaling mechanism.

### 2.2 Resveratrol

Resveratrol (3,40,5‐trihydroxystilbene) (RSV) is found naturally in various foods, especially red grapes, mulberries, red wine, and peanuts. The bioavailability of resveratrol is less than 1% due to its extensive metabolism in the intestine and liver (Walle 2011). Therefore, the intestine has been recommended as a potential target site for its chemopreventive activity (Juan, Alfaras, and Planas 2012). In literary studies, RSV, which significantly affects optimal health, is also accepted as a practical therapeutic component in cancer treatment (Zhang et al. 2013; Ji et al. 2013). A study evaluating the anti‐tumor efficacy of RRL (co‐loading Rapamycin and RSV into liposomes) in HCT116 xenograft mice shows that RRL promotes apoptosis and ferroptosis in tumor cells. This approach represents a promising strategy for treating colorectal cancer (Jia et al. 2024). The impact of RSV on CRC is explained through the mTOR signaling network. This pathway is associated with multiple signaling pathways, including NF‐κB, Wnt, AMPK, and caspases (Vernousfaderani et al. 2021).

In the early stages of CRC, abnormal, mutant APC or APC loss is frequently observed. This pathophysiological process is usually dependent on the Wnt signaling pathway. Activated Wnt stimulates the TSC‐mTOR pathway and causes abnormal proliferation in cancer cells (Wang and Zhang 2014). In the study of Nguyen et al., eight CRC patients were taken with freeze‐dried grape powder, which found 80 g of RSV daily for 14 days. At the end of the research, RSV did not affect CRC cells but blocked the Wnt pathway in normal colon mucosa (Nguyen et al. 2009).

In the multi‐stage carcinogenesis process, RSV phosphorylates AMPK and causes its activation. Afterward, this situation induces autophagy via decreasing the phosphorylated mTOR level. This efficacy is confirmed in the Apc (min) CRC mouse model, where low‐dose RSV was more effective than high‐dose (Cai et al. 2015). The effect of RSV on autophagy is also via the upregulation of Sirt1 in various cell lines (Elshaer et al. 2018; Ma et al. 2022; Zhang et al. 2013). According to studies conducted to understand the mechanism of action of RSV in CRC, NFĸB suppresses growth in the HCT116 cell line by providing Sirt1‐dependent inhibition (Elshaer et al. 2018). Another mechanism for RSV‐induced autophagy induction occurs through telomerase inhibition. High concentrations of RSV induce apoptosis in HT‐29 and WiDr colon cancer cell lines via inhibiting telomerase activity (Dogan and Biray Avci 2018; Elshaer et al. 2018).

The insulin growth factor (IGF), platelet‐derived epidermal growth factor (PDGF), epidermal growth factor (EGF), and vascular endothelial growth factor (VEGF) are bound to their particular receptors. Then, the mTOR signaling cascade is activated under optimal conditions (Khan et al. 2020). The ginkgetin and RSV suppress the angiogenic characteristics of VEGF. Moreover, the synergistic effect of these bioactive ingredients in suppressing endothelial cell proliferation, invasion, migration, and tube formation is confirmed in the HT‐29 cell xenografts in nude mice (Hu et al. 2019).

Studies support the role of elevated mTORC1 and mTORC2 activity in modulating epithelial‐mesenchymal transition (EMT), metastasis, and motility in CRC cells (Gulhati et al. 2011). EMT is a significant event in the first step of the metastatic cascade (Elshaer et al. 2018). RSV reduces EMT‐related signaling factors in CRC cells (Honari et al. 2019) and inhibits the motility and invasiveness of cancer cells (Elshaer et al. 2018).

Cyclic adenosine 3′,5′‐monophosphate (cAMP) level in cancer cells also affects the Akt/mTOR pathway. According to research by Kim et al. (2019) the RSV‐Forskolin combination boosts cAMP levels, which in turn decreases the viability of the DLD‐1 colon cancer cell line by down‐regulating the Akt/mTOR/Myc axis in conjunction with elevated levels of cAMP. In addition, resveratrol suppresses mTORC1 and promotes AMPK activity, leading to indirect pyrimidine synthesis inhibition. Disturbance of pyrimidine nucleotide balance may affect the translation of CRC cells (Amintas et al. 2022).

Abnormalities in metabolic and biochemical processes frequently appear in cancer cells. For example, there is excessive glycolysis and pentose phosphate pathway (PPP) enzyme activation in CRC cell metabolism compared to healthy cells. In this context, pharmacological suppression of the mTOR‐PPP axis may be a promising therapeutic strategy in treating CRC. According to the study of Shibuya et al. (2015) RSV inhibited aerobic glycolysis and PPP in various CRC cell lines. In another study examining CRC metabolism, OSU‐CG5, an energy restriction mimicking agent, was applied to cancer cell lines in a dose‐dependent manner. As a result, OSU‐CG5 reduced p‐mTOR and p‐p70S6K protein production in HCT‐116 and CaCo‐2 cells. Researchers have claimed that energy restriction in cancer cells causes anticancer activity. Interestingly, similar results were obtained with dose‐dependent resveratrol administration (Arafa et al. 2014).

Finally, Raf‐1 kinase inhibitor protein (RKIP) is a tumor cell metastasis inhibitor for survival in various cancers, including CRC. RKIP shows this effect by inhibiting mRaf‐1, PI3K, and MAPK pathways. RSV induces RKIP protein expression in CRC (Dariya et al. 2020).

### 2.3 Fisetin

Fisetin (3,7,3′,4′‐tetra hydroxyflavone) is a polyphenol. Numerous fruits and vegetables, such as apples, strawberries, grapes, dates, cucumbers, and onions, include fisetin (Arai et al. 2000). As a bioactive compound, fisetin has antiproliferative, anticancer, neuroprotective, and antioxidant activity (Haddad et al. 2006; Maher, Akaishi, and Abe 2006; Pal et al. 2013; Khan et al. 2012; Suh et al. 2010; Syed et al. 2013). Fisetin shows therapeutic efficacy in CRC via suppressing the PI3K/Akt/mTOR signaling, and fisetin combination with 5‐FU decreased the total number of intestinal tumors in the treatment of PIK3CA‐mutant CRC (Khan et al. 2019). It also stimulates apoptosis in various cell lines, such as colorectal, prostate, and cervical, by activating the caspase‐3 cascade (Afroze et al. 2022; Syed et al. 2008).

### 2.4 Quercetin

Quercetin is a well‐characterized polyphenolic flavonoid phytochemical with antioxidant activity. According to reports published by various authors, the average daily intake of quercetin is about 25 mg (Nishimuro et al. 2015; Ovaskainen et al. 2008; Pérez‐Jiménez et al. 2011; Reyes‐Farias and Carrasco‐Pozo 2019; Stavric 1994). Onions, asparagus, nuts, and other fruits and vegetables include sufficient amounts (Bhagwat and Haytowitz 2015; Dabeek and Marra 2019). Quercetin shows its therapeutic effect on CRC by modulating signal cascades, involving PI3K/Akt/mTOR, Wnt/β‐catenin, MAPK/ JNK, MAPK/Erk, MAPK/p38, NF‐κB and p‐53 (Arts et al. 2004; Atashpour et al. 2015; Bruning 2013; Howells et al. 2019; Russo et al. 2012; Shi, Tian, and Tian 2021; Zhai et al. 2022). According to that, the application of quercetin to HCT‐116 cells increases the intracellular ROS level. It induces apoptosis through the Sestrin 2/5' AMP‐activated protein kinase (AMPK)/mTOR pathway (Kim, Lee, and Kim 2013).

### 2.5 Ellagic Acid

Ellagic acid (EA) is a natural phenolic component found in grapes, berries, strawberries, blackcurrants, pomegranates, raspberries, green tea, Eucalyptus maculata, Eucalyptus globulus, and ellagitannins in the stems and skins of nut (Girish and Pradhan 2008). EA shows antiproliferative features against various cancer types, including liver, colorectal, breast, prostate, and bladder cancers (Girish and Pradhan 2008; Kao et al. 2012; 2012; Wang, Wang, et al. 2012; Vanella et al. 2013; Ceci et al. 2016). In addition, EA has a therapeutic effect on chronic ulcerative colitis, and it also blocks the development of CRC because of its anti‐inflammatory effects (Kunzmann et al. 2015; Marín et al. 2013; Zhang et al. 2014). The PI3K/Akt signaling has a central role in tumorigenesis in the colon. EA may block colorectal tumorigenesis by inhibiting p‐Akt (Ni et al. 2023).

### 2.6 Apigenin

Apigenin (4′,5,7‐dihydroxyflavone) is a natural flavonoid. It is commonly found in parsley, oranges, tea, and chamomile. It has significant antioxidant, anti‐inflammatory, antiplatelet, and antitumor properties. Moreover, it suppresses the growth and proliferation of cancer cells, promotes apoptotic cell death, stimulates autophagy, induces cell cycle arrest, and disrupts the mitochondrial membrane potential of cancer cells (Yang, Pi, and Wang 2018; Daneshvar et al. 2023). It inhibits the growth of CRC as related to these physiological processes (Chunhua et al. 2013).

The effect of apigenin on CRC occurs by inhibiting the PI3K/Akt/mTOR pathway, which is considered an important therapeutic target due to its abnormal activation in many cancers (Sain, Kandasamy, and Naskar 2022). The Akt/mTOR pathway regulates autophagy, and the apigenin downregulates the Wnt‐ß catenin signaling pathway with the autophagy‐mediated lysosomal degradation system. In this context, Lin et al. have demonstrated the role of the Akt/mTOR signaling pathway in the apigenin‐induced auto‐lysosomal degradation of β‐catenin. The researchers incubated HCT‐116 cells with different concentrations of apigenin. While apigenin treatment reduces p‐Akt, it does not affect total Akt levels. In addition, apigenin exposure reduced the grades of two mTOR downstream substrates, p‐p70S6K and p‐4E‐BP1. As a result, apigenin‐mediated downregulation of β‐catenin occurred through inhibition of the Akt/mTOR signaling pathway (Lin, Chen, et al. 2017). Apigenin was confirmed to inhibit p‐mTOR, p‐PI3K, and p‐Akt expression in HT‐29 cells in a dose‐dependent manner in another study by Chen et al. (2019). Finally, the apigenin glycoside isovitexin decreased the expression levels of p‐PI3K, p‐Akt, p‐mTOR, and Bcl‐2 and significantly increased the levels of Bax and caspase‐3 in human colon cancer epithelial cells (Zhu, Zhao, and Jiang 2021).

### 2.7 Luteolin

Luteolin (3′,4′,5,7‐tetrahydroxyflavone) flavonoids are commonly found in high concentrations in fruits and vegetables. Increasing evidence suggests that luteolin has antioxidant, anti‐inflammatory, and antitumor characteristics in many cancers (Farooqi et al. 2020; Imran et al. 2019; Pu et al. 2018). It is an undisputed fact that one of these cancers is CRC. In this context, the vanadium‐luteolin complex applied in a dose‐dependent manner induced apoptosis in the HT‐29 cell line in the study of Roy et al. As a result, the p53‐mediated activation of vital proteins in intrinsic pathways, including caspase‐3, Bax, Bcl2, and mTOR/Akt, was in charge of this therapeutic benefit (Roy and Chakraborty 2018). In a different study, the expression of Bcl‐2, mTOR, and Akt dramatically reduced when treatment of luteolin and quercetin with 5‐Fluorouracil (5‐FU) in the HT‐29 cells. Moreover, the combined treatment also caused an increase in the expression levels of pro‐apoptotic proteins such as PTEN, p53, Bax, and p38 MAPK. However, the treatment of 5‐FU alone did not have a similar effect on mRNA and protein levels of mTOR compared to the combined treatment (Erdoğan, Ağca, and Aşkın 2022).

### 2.8 Silibinin

Silibinin is an active ingredient extracted from the seeds of milk thistle *Silybum Marianum*. Traditionally, silibinin has beneficial effects in liver diseases. Moreover, it has been used to treat several cancers due to its inhibitory effect on cancer cells' growth, proliferation, and angiogenesis (Sameri et al. 2021; Sayyed, Heuertz, and Ezekiel 2022). In this regard, silibinin has a preventative impact on colon cancer brought on by azoxymethane (Sameri et al. 2021). According to Raina and Agarwal's study, silibinin's anti‐CRC effects are related to inhibition of the Wnt/β‐catenin pathway, which is known to play a significant role in the onset and progression of CRC (Raina and Agarwal 2013). Silibinin inhibits the formation of EMT and cancer stem cells, which play a role in cancer relapse through the downregulation of beta‐catenin. At the same time, silibinin applied to HCT‐116 cells induces apoptosis by leading to the upregulation of Bax, a pro‐apoptotic protein, and downregulation of Bcl‐2, an anti‐apoptotic protein (Sameri et al. 2021). On the other hand, mechanistic studies revealed that silibinin is a potent inhibitor of the PI3K/Akt/mTOR pathway (Belli et al. 2017; Raina et al. 2013; Raina and Agarwal 2013). PRKAA2 functions as a fuel sensor. PRKAA2, activated by phosphorylation due to decreased cellular ATP levels and the concomitant increase in AMP levels, inhibits mTOR activity‐related anabolic pathways. Silibinin causes energy limitations in CRC cells by significantly activating PRKAA2 (Raina et al. 2013). It activates PTEN in the PTEN/Akt pathway, one of the mTOR‐related apoptotic pathways, and reduces the production of p‐Akt (Tsai et al. 2015). Additionally, silibinin suppresses the development of CRC stem cells by blocking the PP2Az/AKT Ser473/mTOR pathway (Wang, Chang, et al. 2012). These findings suggest that silibinin may be beneficial in improving CRC‐associated morbidity and mortality (Raina et al. 2013).

### 2.9 Isorhamnetin

Isorhamnetin is found in pears, apples, and blackberries. It is also a predominant plasma metabolite of quercetin (Li et al. 2014). As a bioactive ingredient, it has broad antitumor activity, inhibiting human cervical, lung, pancreatic, colon, breast, liver, stomach, nasopharyngeal, and other cancer cells. In this context, it suppresses the proliferation of tumor cells, induces apoptosis, and regulates tumor suppressor genes, proto‐oncogenes, and signaling pathways. It also activates the mitochondrial signaling pathway of apoptosis (Gong et al. 2020; Hu et al. 2015).

Isorhamnetin triggers an inhibitory effect on Akt/mTOR and MEK/Erk signaling pathways in breast cancer, which are closely related to cell proliferation and survival (Hu et al. 2015). In a study conducted by (Liu 2011), isorhamnetin induces cell cycle arrest in the G2/M phase in HT‐29, HCT‐116, and SW480 cell lines and inhibites cell proliferation by inhibiting the PI3K/Akt/mTOR pathway (Jaramillo et al. 2010; Li et al. 2014).

### 2.10 Anthocyanin

Anthocyanins/anthocyanidins (A/A) contain more than 500 water‐soluble compounds. They are found naturally in high amounts in many colorful fruits, vegetables, leaves, and flowers (de Sousa Moraes et al. 2019). They contribute to the survival rate and improve the quality of life in patients with CRC because of their antioxidant and anti‐inflammatory properties (de Sousa Moraes et al. 2019). The molecular mechanism of anthocyanins occurs via suppressing pro‐inflammatory pathways by downregulating MMPs before metastasis. MMPs positively correlate with the Akt/mTOR pathway in CRC cells. Studies have shown that pomegranate juice, lyophilized strawberries, and red grape extract, which are known to have high anthocyanin content, cause activation of AMPK and inhibition of MMPs and Akt/mTOR signaling pathway in vivo and in vitro CRC models. This situation reduces the invasive phenotype in CRC. Therefore, they have a significant role in inhibiting metastasis in CRC (Banerjee et al. 2013; de Sousa Moraes et al. 2019). They significantly reduce p‐mTOR compared to rapamycin, a synthetic mTOR suppressor. However, the anthocyanin‐induced inhibition of mTOR can be entirely reversed by deactivating AMPK‐1. When AMPK‐1 is inactivated, anthocyanins lose their ability to inhibit mTOR in the HT‐29 cells (Lee et al. 2010).

## 3 The Potential Role of Terpenoids

**FIGURE 3:**
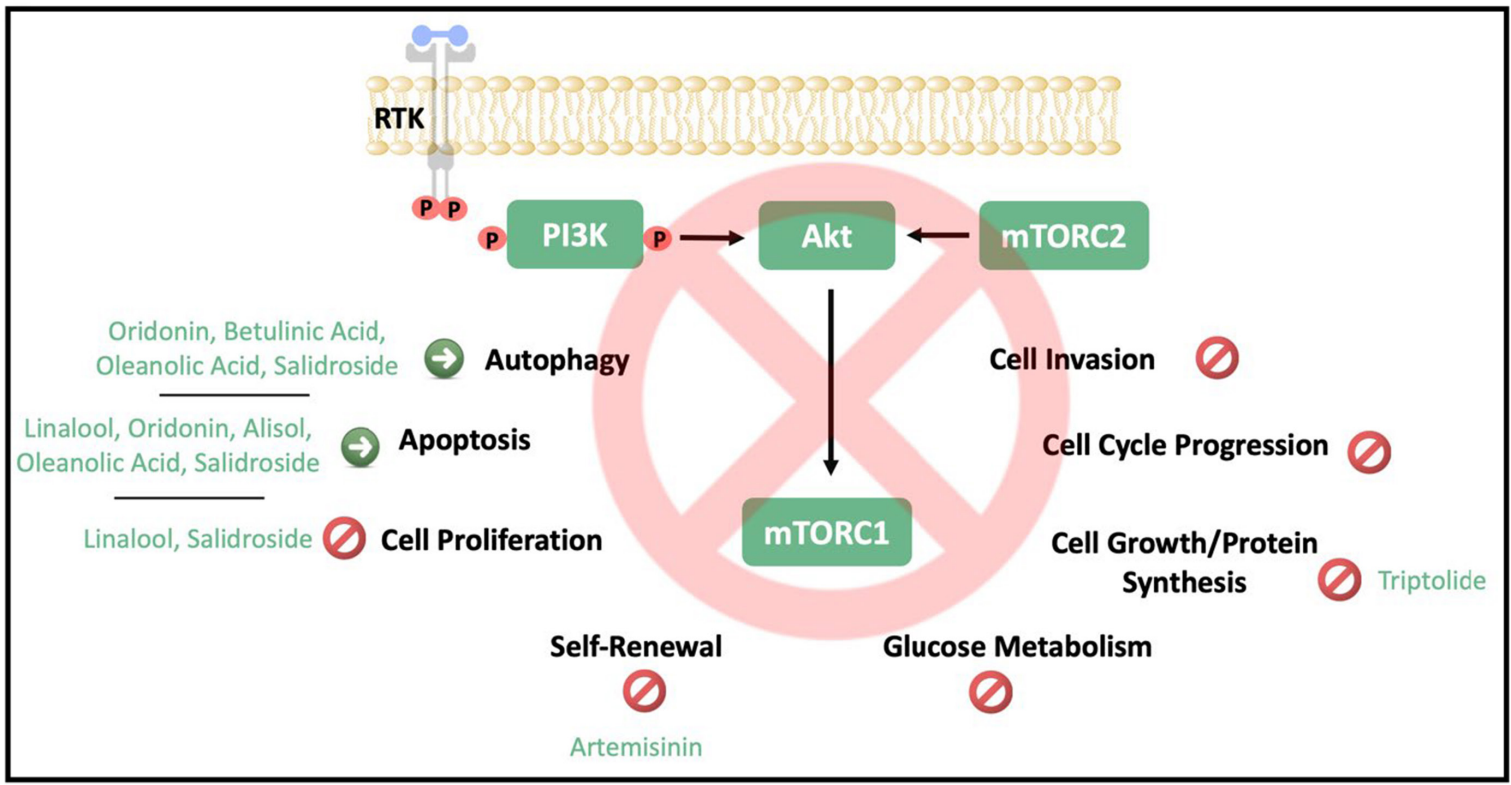
The cellular effects of terpenoids on CRC progression. P, Phosphate; RTK, Receptor tyrosine kinase.

Terpenoids are the main compounds of essential oils found in marine organisms, fungi, plants, and some animals. They include many compounds with different structures and properties and are synthesized in plants differently (Galindo‐Solís and Fernández 2022). Most terpenoids' localizations are the fruits, leaves, seeds, flowers, roots, and stems of plants (El‐Baba et al. 2021). The roles of terpenoids as pharmaceutical active ingredients are antioxidant, anti‐inflammatory, antibacterial, antiviral, and antifungal features. Moreover, some terpenoids have antitumor activity in cancer cells because of induction of apoptosis and cell cycle arrest. However, these studies are still in the research phase, and further molecular analysis is required (Bergman, Davis, and Phillips 2019). This section discusses alkaloids' potential therapeutic role in CRC through inhibition of the PI3K/Akt/mTOR pathway, and findings are summarized in Table 2. Additionally, the cellular effects of terpenoids via PI3K/Akt/mTOR signaling in CRC are shown in Figure 3.

**TABLE 2:** According to results of in vitro studies, the effects of various terpenoids on the CRC progression via mTOR inhibition.

Bioactive compound	Study design	Effects via mTOR inhibition	References
Linalool	Treatment of linolool on the HCT116 and SW480 cells	Modulation of proliferation, apoptosis, malignancy, and cell invasion ability in CRC cells	Hou et al. (2022)
Oridonin	Treatment of oridonin on the DLD1 cells	Induction of apoptosis and autophagy of colon cancer DLD‐1 cells via regulating the AMPK/mTOR/ULK1 pathway	Bu et al. (2020)
Triptolide	Treatment of LLDT‐246, a new triptolide derivative on the HCT‐116 cells	Indirectly affects NF‐κB signaling primarily by modulating AKT/GSK3β/mTOR	Li et al. (2014)
Alisol	Dose‐dependent Alisol A treatment on the HCT‐116 and HT‐29 cell lines	Reduce viability and induce apoptosis in CRC cells and also prevention of CRC progression	Han et al. (2022)
Betulinic Acid	Treatment of betulinic acid on the HCT‐116, SW‐480 CRC and cell lines and NCM‐460 normal colonic epithelial cells	Induction of a protective autophagy by inhibiting Akt/mTOR signaling	Wang et al. (2017)
Oleanolic acid	OA treatment on the HCT‐116 and SW‐480 CRC cell lines and NCM‐460 normal colonic epithelial cells	Induction of autophagy and apoptosis through AMPK activation and mTOR suppression	Hu, Cao, et al. (2021),
Salidroside	Dose‐dependent treatment of salidroside on the HT‐29 cells	Triggering apoptosis and autophagy and exhibition of potent anti‐proliferative properties through suppression of the PI3K/Akt/mTOR signaling pathways	Fan et al. (2016)
	Treatment salidroside alone or in conjunction with chemotherapeutic drugs on HCT‐116 cells	Induction of autophagy via regulation AMPK/mTOR signaling pathway (up‐regulation p‐AMPK, down‐regulation p‐mTOR in CRC cells)	Li and Chen (2017)

### 3.1 Linalool

Linalool is an acyclic monoterpenoid alcohol that imparts fragrance and aroma in cosmetics, soaps, and perfumes. Linalool has antioxidant, anti‐inflammatory, anxiolytic, anticonvulsant, analgesic, and antipsychotic effects. Moreover, recent research has shown that linalool is an effective agent against CRC. It induces oxidative stress by increasing free radicals in CRC cells. In vitro and in vivo studies indicate that linalool‐induced oxidant stress causes lipid peroxidation, apoptosis, and DNA damage in CRC cells (Iwasaki et al. 2016; Thapa et al. 2019). This situation is specific to cancer cells. Therefore, linalool can be a potential candidate for use in the treatment of colon cancer. The potential mechanism is the modulation of Akt/mTOR and JAK2/STAT3 pathways. Activation of these pathways leads to inhibition of cell migration, cell viability, and apoptosis (Donadu et al. 2017; Hou et al. 2022).

### 3.2 Artemisinin

Artemisinins (Art) are sesquiterpene lactones containing a peroxide moiety. It is extracted from the plant *Artemisia annua* (Cheong et al. 2020). Art, generally used to treat malaria, has anti‐inflammatory, antioxidant, and anticancer effects. Although its anticancer activity, Art's low bioavailability, low solubility, and short half‐life make it challenging to use as a drug in cancer treatment. Applying nanotechnology‐based strategies may be a promising technique to overcome these disadvantages. Thus, the activity of the bioactive compound in target cells and its transfer to these cells can be increased (Firouzi Amandi et al. 2023).

In the paper of Huang et al. (2022) Artesunate, a derivative of Art, was applied to SW480 and HCT116 CRC cell lines. The researchers found that Artesunate causes excessive ROS production, inhibits cell proliferation, and affects autophagy in CRC cells. Other studies revealed the efficacy of dihydroartemisinin (DHA), an Art metabolite, against CRC. In this context, DHA significantly blocked cancer stem‐like cells (CLCSs) properties in CRC through inhibition of Akt/mTOR signaling (Wang et al. 2022, 2023).

### 3.3 Oridonin

Oridonin is an ent‐kaurene diterpenoid compound. It is extracted from the Chinese medicinal plant *Rabdosia rubescens*. The pharmacological and physiological functions of oridonin are antibacterial, anti‐inflammatory, and anticancer features (Xu, Liu et al. 2015). Its anticancer effects in CRC can be emerged by stimulation of the bone morphogenetic protein 7(BMP7)/p38 mitogen‐activated protein kinases (p38MAPK)/p53 or p38MAPK/phosphatase & 10 sin homolog (PTEN) signaling pathways (Liu et al. 2018; Wu et al. 2016). In addition, oridonin induced apoptosis and autophagy in DLD‐1 cells by modulating the AMPK/mTOR/ULK1 (Bu et al. 2020). However, although oridonin can inhibit the growth of tumor cells in various cancer types, the specific cellular targets of oridonin‐induced cytotoxicity in colon cancer cells have not been adequately studied (Bu, Shen, and Cui 2019).

### 3.4 Triptolide

Triptolide (TP), a diterpene trioxide, is an essential active compound of extracts derived from *Tripterygium wilfordii* Hook F (TWHF) (Hu et al. 2014). TP is widely used in treating inflammatory and autoimmune diseases, tumors, and organ transplantation (Chen et al. 2021). In this context, in a study examining the function of TP in the progression of inflammatory bowel disease, TP was found to induce classically activated macrophages (M1) to alternatively activated macrophages (M2) repolarization of RAW 264.7 macrophages via mTOR/signal transducer and activator of transcription 3 (STAT3) signaling. Thus, it attenuates lipopolysaccharides' (LPS) induced activation (Zhu et al. 2022). However, the clinical application of TP is restricted due to its severe toxicity (Liu 2011). LLDT‐246, a novel triptolide derivative, exhibited a slightly more potent NF‐κB inhibition and cytotoxicity activity on HCT‐116 cells than its maternal compound. Furthermore, LLDT‐246 shows low toxicity in healthy cells. The molecular mechanism of this physiological process is related to Akt inhibition, glycogen Synthase Kinase 3β (p‐GSK3β), and p‐mTOR. However, 70 kDa heat shock proteins (HSP70) did not have a significant effect on phospho‐extracellular receptor kinase (p‐ERK) and phosphorylated JNK (p‐JNK) levels. LLDT‐246 indirectly affects NF‐κB signaling primarily by modulating AKT/GSK3β/mTOR (Li, Wang et al. 2014). To sum up, LLDT‐246 is a promising anticancer derivative of TP. Nevertheless, more molecular studies on its detailed mechanism of action are needed.

### 3.5 Alisol

Alisol is extracted from the rhizomes of the *Alisma orientale*. It has anti‐metabolic, antiviral, antibacterial, anti‐inflammatory, immunomodulatory, and anticancer activities. Moreover, it has been established to be effective in prostate, breast, stomach, liver, colon, and ovarian cancers (El‐Baba et al. 2021). According to Han et al. study, dose‐dependent Alisol A treatment on the HCT‐116 and HT‐29 cells decreased CRC proliferation, induced cell cycle arrest, and apoptosis. Alisol A inactivates the PI3K/Akt signaling pathway and prevents CRC progression by suppressing the phosphorylation levels of PI3K, Akt, and mTOR in CRC cells. Further studies are required to understand the action mechanism of Alisol A in CRC (Han et al. 2022).

### 3.6 Betulinic Acid

Betulinic acid (BA) is a pentacyclic triterpenoid. Its chemical structure consists of rings and side chains containing a carboxylic acid and a hydroxyl group. The molecular mechanism of BA's anticancer activity needs to be clarified. However, Wang et al. revealed that BA treatment induced a dose‐dependent cytotoxic impact in CRC cells. This effect is related to the autophagy mechanism triggered by Akt/mTOR pathway inhibition (Wang et al. 2017).

### 3.7 Oleanolic Acid

Oleanolic acid (OA) and its derivatives are isolated from *Olea ferruginea*. The biological activities of OA derivatives in CRC are cytotoxic, antioxidant, anti‐inflammatory, antimicrobial, anti‐angiogenic, and immunomodulatory effects (Sultana and Ata 2008). In the literature, various studies reported that nearly all these effects emerged mediated by the mTOR signaling pathway (Gao et al. 2010, 2011; Hu, Cao, et al. 2021). Hu et al. have demonstrated that OA treatment in HCT‐116 and SW‐480 cell lines induces autophagy and apoptosis mechanisms and suppresses proliferation and viability. This anticancer activity appeared via activating AMPK and inhibiting mTOR (Hu, Cao, et al. 2021). Apart from natural compounds of OA, the effects of oleanane synthetic triterpenoids in CRC are investigated in vitro molecular analysis. Similarly, findings confirmed that synthetic oleanane derivatives suppress the mTOR pathway in CRC cell lines (Gao et al. 2010, 2011).

### 3.8 Salidroside

Salidroside is a phenylpropanoid glycoside. It is extracted from *Rhodiola rosea* L., known as “golden root” (Fan et al. 2016; Rong et al. 2020). Salidroside shows antitumor function against gastric, colon, breast, and bladder cancer and anti‐hypoxia, antiaging, and immunomodulatory effects as a medicinal plant (Rong et al. 2020). Research conducted to determine the mechanism of action of salidroside on CRC cells is remarkable. In this regard, a study performed by Fan et al. has shown that salidroside exhibited potent antiproliferative action in the HT‐29 cell line by dose‐dependently triggering apoptosis and autophagy. Additionally, salidroside treatment causes decreased gene expression of p‐Akt, p‐PI3K, and p‐mTOR (Fan et al. 2016). Li and Chen (2017) discovered that salidroside exhibited identical molecular effects when administered to HCT‐116 cells alone or in conjunction with chemotherapeutic drugs.

## 4 The Potential Role of Alkaloids

**FIGURE 4:**
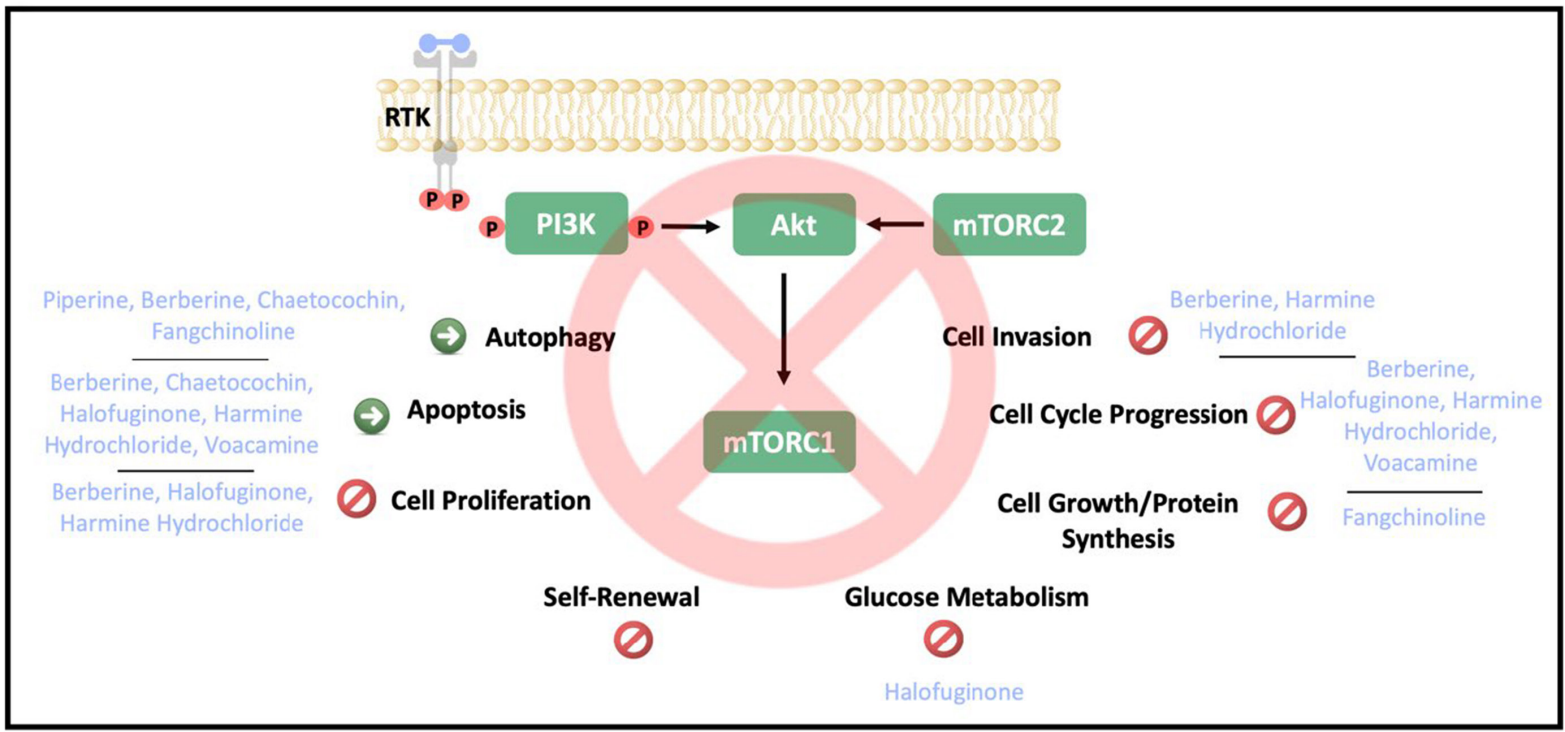
The cellular effects of alkaloids on CRC progression. P, Phosphate; RTK, Receptor tyrosine kinase.

Alkaloids are unique and valuable compounds for drug discovery. W. Meissner discovered alkaloids in 1818 (Preininger 1975). The following studies showed the presence of nitrogen atoms in a heterocyclic ring system in their structure. The classifications of alkaloids are primary, secondary, tertiary, or quaternary amines. Alkaloids are found mainly in herbs, humans, animals, fungi, and other microorganisms (Dewick 2009). Alkaloids have antiproliferative, antiviral, antibacterial, antimetastatic, and insecticidal activity on various cancers (Qıu et al. 2014). This section discusses alkaloids' potential therapeutic role in CRC through the PI3K/Akt/mTOR pathway, and beneficial effects are given in Table 3. Moreover, the cellular effects of alkaloids via PI3K/Akt/mTOR signaling in CRC are presented in Figure 4.

**TABLE 3:** According to results of in vitro studies, the cellular effects of various alkaloids on the CRC progression via mTOR inhibition.

Bioactive compound	Study design	Effects via mTOR inhibition	References
Piperine	Piperine treatment alone and in combination with curcumin on the Caco‐2 and HT‐29 CRC cells	Implications for tumorigenesis and inflammation	Moreau and Kaur (2017)
Berberine	Berberine treatment on the HCT116, SW480, and LOVO cells	Prevention of the progression of cancer via inhibition of tumor multiplicity and tumor growth	Li et al. (2015)
	Berberine treatment on the SW480 cells	Inhibition and promotion the expression of the upstream proteins (HDACs and PTEN, respectively), inhibition of cell proliferation, induction of apoptosis and autophagy, depolarization of mitochondrial membrane potential, and arrestion G1 phase of cell cycle	Li et al. (2021)
	Berberine and oligomeric proanthocyanidins synergetic treatment on the RKO and HT29 human CRC cell lines	Regulation of the oncogene expression and inhibition of cell proliferation, colony formation, migration, and ınvasion through enhanced cell apoptosis	Okuno et al. (2022)
	Berberine treatment on the HT‐29, SW‐480 and HCT‐116 cells	Inhibition growth, migration, and invasion of these colon cancer cell lines via down‐regulation of aquaporins expression and up‐regulating phosphatase and tensin (PTEN) and regulating tumor metastasis	Tarawneh et al. (2023)
Chaetocochin	Chaetocochin J treatment on the SW480, HCT116 and RKO cells	Regulation of downstream signaling cascade, induction of apoptosis and autophagy simultaneously in SW480 and RKO cells	Hu et al. (2021b)
Halofuginone	Halofuginone treatment on the HCT116, SW480, SW620, HT29, DLD‐1, IEC‐6 and MIHA cells	Slows glycolysis and inhibits lipid biosynthesis in HCT116 and SW480 cells, inhibition cell proliferation through arresting of G1/G0 cell cycle progression, induction the generation of reactive oxygen species while reduction of NADPH production, and induction of apoptosis in HCT116 and SW480 cells	Chen et al. (2015)
	Halofuginone treatment on the HCT116 and SW480 cells	Activation ULK1 by downregulation of its phosphorylation site at Ser757and induction of autophagic flux under nutrient‐rich condition, formation of autophagosome is blocked in nutrient‐poor environment, inhibition glycolysis under nutrient‐rich condition and inhibition gluconeogenesis under nutrient‐poor condition	Chen et al. (2017)
Harmine hydrochloride	Harmine hydrochloride treatment on the HCT116 cells	Inhibition cell proliferation and induction cell migration and apoptosis by regulating expression of the Bcl‐2 family genes and mitochondrial proteins	Kim (2021)
Fangchinoline	Fangchinoline treatment on the HT29 and HCT116 cells	Promotes cell autophagy, inhibition CRC cell growth, induction protective autophagy	Xiang et al. (2021)
Voacamine	Voacamine treatment on the CT26 and HCT116 cells	Induction of the late‐stage apoptosis in CRC cells, proliferation and migration of CT26 and HCT116 cells, suppression tumor cell colony formation, inhibition of cells arrest in G2‐M and G0‐G1 phases, distruption of mitochondrial membrane potential	Chen et al. (2022)

### 4.1 Capsaicin

Capsaicin is a secondary metabolite responsible for the characteristic taste of the Capsicum genus. Capsaicin and its analogs have been used for medical purposes for centuries. Recent researches have shown the antioxidant, analgesic, anti‐inflammatory, anti‐obesity, and anticancer properties of capsaicin (Chapa‐Oliver and Mejía‐Teniente 2016). In particular, the antitumor activity of capsaicin has been investigated on several cancer cell lines (Lin, Wang, et al. 2017; Dai et al. 2018; Que et al. 2022). Its underlying mechanism has remained unknown.

On the other hand, STAT‐3 regulates the apoptosis‐related gene expression and abnormal activation of STAT‐3 in colorectal carcinomas (Gargalionis, Papavassiliou, and Papavassiliou 2021). Targeting STAT3 signaling is suggested as an efficient approach for CRC treatment (Lee, Jeong, and Ye 2019). Consistently, Yang et al. have revealed that Akt/mTOR and STAT‐3 signaling mechanisms are essential modulators of metastasis in CRC by depending on capsaicin concentration. They showed that the capsaicin treatment promoted metastasis and invasion by triggering ROS production and regulating Akt/mTOR and STAT‐3 pathways. Unlike the other bioactive compounds, capsaicin exhibits mutagenic effects through Akt/mTOR and STAT‐3 signaling mechanisms and promotes tumorigenesis (Yang et al. 2013).

### 4.2 Piperine

Piperine is an alkaloid isolated from a few of the Piperaceae family, especially *Piper nigrum* (black pepper) (Smilkov et al. 2019). As a traditional medicine, piperine has numerous bioactivities and pharmacological properties, such as antiseptic, regulator of the digestive system, diuretic, antibacterial, and insecticide activities (Meghwal and Goswami 2013). Recently, it has been focused on anticancer effects in CRC with cell culture and experimental colon cancer models (de Almeida et al. 2020; Duessel, Heuertz, and Ezekiel 2008; Li et al. 2022; Mohammadian et al. 2022; Rehman et al. 2020; Shaheer, Somashekarappa, and Lakshmanan 2020). The main chemopreventive mechanisms of piperine include the activation of apoptotic signaling cascades, inhibition of cell proliferation, changes in redox homeostasis, modulation of ER stress and autophagy, inhibition of angiogenesis, induction of detoxification enzymes, sensitization of tumors to radiotherapy and chemotherapy (Bolat et al. 2020; Duessel, Heuertz, and Ezekiel 2008; Kirubhanand et al. 2020; Rather and Bhagat 2018; Shaheer, Somashekarappa, and Lakshmanan 2020).

Piperine is a substantial negative regulator of autophagy via modulation of mTOR. In this context, piperine inhibits the mTORC1 pathway in Caco‐2 and HT‐29 cells (Moreau and Kaur 2017). In addition, piperine is included in curcumin‐containing formulations as it increases the bioavailability of curcumin (Bolat et al. 2020; Lambert et al. 2004; Shoba et al. 1998).

### 4.3 Berberine

Berberine is an isoquinoline quaternary alkaloid. It is found in various plant species, such as *Hydrastis canadensis, Berberis aristata, Coptis japonica, Coptis chinensis*, and *Phellondendron amurense*. Berberine has several pharmacological properties, including therapeutic effects against gastroenteritis, obesity, fatty liver disease, hypertension, metabolic syndrome, and coronary artery diseases. Moreover, it has antitumorigenic function against many types of cancer, including CRC (Xiong et al. 2022). However, the underlying molecular mechanism in this section remains unknown. In recent years, in vitro analyses conducted on various cancer cell lines have shown that berberine inhibits cancer cells' proliferation and cell migration and induces apoptosis. (Ortiz et al. 2014; Vishnoi et al. 2021). In this context, berberine stimulates caspase‐3 activation in the HCT‐116 cell line and down‐regulates miR‐21 expression while increasing the expression of integrin β4 (ITGβ4) and programmed cell death 4 (PDCD4) proteins (Lü et al. 2018). Additionally, berberine suppresses cyclin D1 and arrests the cell cycle in the G1/G0 phase by enhancing p27 and p21 in HCT‐116 and HT29 cell lines (Zhao et al. 2021). Berberine is a reliable and effective phytochemical for CRC treatment. The therapeutic activity occurs through the organization of gene expression at the transcriptional and translational level, modulation of the cell cycle, regulation of inflammatory and metabolic signaling pathways such as AMPK, JAK2/STAT3, Wnt/β‐catenin, IL‐6/STAT3/NF‐κB, and cyclooxygenase‐2 (COX‐2)/prostaglandin E2 (PGE2) (Jiang et al. 2022).

Okuno et al. (2022) have shown that combining berberine with oligomeric proanthocyanidins gives rise to significant inhibition of Akt in CRC cell lines (Okuno et al. 2022). Essentially, AMPK activation and mTOR inhibition play substantial roles in the effectiveness of berberine on CRC treatment. Li et al. (2021) demonstrated that dose‐dependent berberine treatment induces the *PTEN* gene expression and inhibits the production of PI3K, Akt, and mTOR proteins in SW‐480 cells. In another study, it has revealed that berberine up‐regulates PTEN while down‐regulates Aquaporin proteins in SW‐480, HT‐29, and HCT‐116 cell lines. The excessive expression level of the PTEN inhibits the PI3K/Akt/mTOR pathway and contributes to the anticancer activity of berberine (Tarawneh et al. 2023). Moreover, berberine inhibits colon cancer cell survival by modulating the translocation of β‐catenin from the nucleus to the cytoplasm (Nie et al. 2022). In conclusion, berberine's chemopreventive and tumor‐suppressive properties on CRC occur through multiple mechanisms on the several pathways, such as mTOR, and Wnt‐ β‐catenin (Li et al. 2015; Tarawneh et al. 2023).

### 4.4 Piperlongumine

Piperlongumine, isolated from *Piper longum* L., is an active alkaloid with anti‐angiogenic, anti‐atherosclerotic, antibacterial, anti‐inflammatory, and antitumor properties. According to recent evidence, piperlongumin may be an effective agent that inhibits metastasis and invasion in CRC‐induced mice (Huang et al. 2020). In another study, Kumar et al. revealed that this effect's underlying molecular mechanism is related to the modulation of the Ras/PI3K/Akt/mTOR pathway (Kumar and Agnihotri 2019). In vitro analyses performed on different types of cancer also confirmed these outcomes (Shrivastava et al. 2014; Wang et al. 2015).

### 4.5 Chaetocochin

Chatomium genus mushrooms produce structurally diverse and complex natural compounds. In addition to their toxic effects against pathogens, these compounds harm plants, animals, and humans. Currently, Index Fungorum lists approximately 400 species of chaetomium. (Xu et al. 2015). The first chaetocochin was isolated from the Chaetomium globosum rice culture, a structurally isomeric form of chetomin (Viziteu et al. 2016). Chaetocochin exhibits anticancer activity on several cancer cell lines. Mainly, it suppresses the proliferation of CRC cells and induces apoptosis and autophagy. Regulating the downstream cascades of the AMPK and PI3K/Akt/mTOR signaling pathways increases the antitumor effect (Hu, Yin, et al. 2021).

### 4.6 Halofuginone

Halofuginone is isolated from the Chinese plant *Dichroa febrifuga* Lour (Jin et al. 2014). It has been used as an antioxidant drug in livestock for decades (de Figueiredo‐Pontes et al. 2011). Literature has demonstrated that it can suppress many cancer types, including liver cancer, bladder cancer, melanoma, breast cancer, and leukemia (Huo et al. 2015). Moreover, it suppresses cell proliferation and induces autophagy and apoptosis. Triggering apoptosis is achieved by downregulating of the Akt/mTORC1 pathway. Additionally, it blocks cell growth in CRC cells by reducing glucose uptake and glycolysis according to in vitro and in vivo analyses (Chen et al. 2015). Afterward, the same researchers discovered that halofuginone induces autophagy by downregulating the Akt/mTORC1 signaling pathway only under nutrient‐rich conditions (Chen et al. 2017).

### 4.7 Harmine

Harmine, a β‐carboline alkaloid, is isolated from the seeds of *Peganum harmala*. The *P. harmala* L. plant is a perennial plant from the Zygophyllaceae family. It has a wide scale of pharmacological effects, particularly on the central nervous system, cardiovascular system, musculoskeletal system, and ion channels (Tabrizizadeh et al. 2018). Harmine can inhibit cellular and humoral immunity and monoamine oxidase. Nevertheless, it demonstrates anticancer, anti‐inflammatory, analgesic, antipruritic, and antipsoriasis effects (Filali et al. 2015).

Harmine has an inhibitory effect, particularly on the gastric, cervical, stomach, liver, nasopharyngeal, pancreatic, leukemia, and CRC (Ding et al. 2015; Jiménez et al. 2008). However, it may lead to serious side effects, especially on the central nervous system, such as tremors, convulsion, excitation, and neural inhibition (Louis and Zheng 2010). Therefore, researchers have shifted to the anticancer effects of harmine hydrochloride, a hydrophilic and stable substance similar to harmine but with reduced toxicity. Studies have shown that harmine hydrochloride inhibits the growth, colony formation, and migration ability of HCT116 cells. Additionally, harmine hydrochloride reduces p‐ERK, p‐PI3K, p‐AKT, and p‐mTOR in HCT‐116 cells, and thus it induces apoptosis by inhibiting Erk and PI3K/Akt/mTOR signaling pathways (Kim 2021).

### 4.8 Fangchinoline

Fangchinoline (FAN) is abundantly found in the dried roots of *Stephania tetrandra* S. Moore (Menispermaceae), a plant identified with antioxidant and anti‐inflammatory effects (Tang et al. 2013). In recent times, FAN has mostly gained attention for its anticancer activity (Wang et al. 2011; Tian, Ding, and Li 2015; Yang et al. 2020). FAN inhibits cell viability and cell cycle progression in CRC. In addition, it triggers autophagy by suppressing the AMPK/mTOR/ULK1 signaling mechanism (Jiang et al. 2021). Consequently, FAN, a potent autophagy‐inducing agent, appears to be a substantial component of CRC treatment (Xiang et al. 2021).

### 4.9 Voacamine

Voacamine is a typical compound of bisindole alkaloids and it is extracted from the Voacanga species (Apocynaceae) (La Barre, Lequime, and Van Heerswynghels 1955). Voacamine synergizes with other antitumor drugs in different cancer types in cell apoptosis and suppression of the growth of cancer cells (Lee et al. 2015). Voacamine promotes autophagic cell death independent of apoptosis, especially in multidrug‐resistant human cancer cells (Condello et al. 2020). These findings prove that voacamine is a potential therapeutic agent for integrative oncological treatments against resistant tumors (Condello et al. 2014). A study evaluating the anticancer activity and potential mechanisms of voacamine against CRC cells has shown that it inhibited the proliferation and cell migration of CT26 and HCT116 cells. Voacamine markedly disrupts the mitochondrial membrane potential and elicits mitochondrial dysfunction in CRC. Thus, the relevant pro‐apoptotic proteins reach high expression levels and concomitant intracellular ROS accumulation. Additionally, it suppresses the EGFR/PI3K/Akt pathway and inhibits CRC development by inducing the transcription factors EGFR, PI3K, p‐mTOR, p‐Akt, p‐NF‐kB, and p‐P70S6. These findings have implicated voacamine as a potential therapeutic agent for treating CRC (Chen et al. 2022).

## 5 Conclusion

The therapeutic activity of nearly all investigated bioactive compounds on CRC has been related to PI3K/Akt/mTOR signaling suppression. In this context, the underlying assumption is that blockage of abnormal PI3K/Akt/mTOR signaling in the CRC cells can provide therapeutic effects in the CRC progression. However, each bioactive component causes various physiological processes in CRC lines. They are free radical production, disruption of mitochondrial membrane potential, and blockage of cancer stem cell migration, cell cycle, and glycolysis. As a result, carcinogenesis is inhibited by stimulating autophagy and apoptosis. However, it should be noted that most of the studies consist of in vitro dose‐dependent treatment studies. mTOR signaling is present in almost all cells and is responsible for different physiological functions. The applied bioactive component should act specifically on the cell.

On the other hand, various in vitro studies have evaluated the impacts of many bioactive components and chemotherapy combinations on CRC treatment. Most bioactive compounds were treated in limited doses, and such approaches make it difficult to determine the effective dose for inhibition of the mTOR pathway. While evaluating the bioactive components' PI3K/Akt/mTOR signaling pathway, their combined use with existing pharmacological agents should be examined in clinical trials. Moreover, integrative omic studies should investigate the alteration of the mTOR signaling pathway activation in healthy cells. In addition, the potential of phenolic compounds to overcome drug resistance in CRC should be examined.

Apart from that, delivery systems of bioactive compounds are also vital in determining the practical value. Liposomes may be preferred to increase the bioavailability of specific bioactive components, such as curcumin and RSV. mTOR inhibition by applying bioactive compounds using advanced drug delivery techniques may contribute to CRC treatment in further in vivo studies.

## Author Contributions

**Kübra Demir:** conceptualization (lead), investigation (equal), resources (equal), writing – original draft (equal). **Rana Turgut:** conceptualization (equal), investigation (equal), resources (equal), writing – original draft (equal). **Selcen Şentürk:** conceptualization (equal), investigation (equal), resources (equal), writing – original draft (equal). **Handan Işıklar:** conceptualization (supporting), investigation (equal), resources (supporting), writing – original draft (supporting). **Elif Günalan:** supervision (lead), writing – review and editing (equal).

## Ethics Statement

The authors have nothing to report.

## Conflicts of Interest

The authors declare no conflicts of interest.

## Data Availability

The authors have nothing to report.
